# A call to action: informing research and practice in suicide prevention among individuals with psychosis

**DOI:** 10.3389/fpsyt.2024.1378600

**Published:** 2024-04-22

**Authors:** Samantha A. Chalker, Roxanne Sicotte, Lindsay A. Bornheimer, Emma M. Parrish, Heather Wastler, Blaire Ehret, Jordan DeVylder, Colin A. Depp

**Affiliations:** ^1^ Veterans Affairs San Diego Healthcare System, San Diego, CA, United States; ^2^ Department of Psychiatry, University of California, San Diego, CA, United States; ^3^ Department of Psychiatry and Addictology, Faculty of Medicine, Université de Montréal, Montréal, QC, Canada; ^4^ Research Center of the Centre Hospitalier de l’Université de Montréal (CRCHUM), Montréal, QC, Canada; ^5^ University of Michigan, School of Social Work, Ann Arbor, MI, United States; ^6^ Department of Psychiatry, University of Michigan Medical School, Ann Arbor, MI, United States; ^7^ San Diego State University/University of California San Diego Joint Doctoral Program in Clinical Psychology, San Diego, CA, United States; ^8^ Department of Psychiatry & Behavioral Health, The Ohio State University Wexner Medical Center, Columbus, OH, United States; ^9^ Lyra Health, Burlingame, CA, United States; ^10^ Silver School of Social Work, New York University, New York, NY, United States

**Keywords:** suicide, psychosis, suicide behavior, psychotic symptoms, schizophrenia

## Abstract

Although it is well established that individuals living with psychosis are at increased risk for suicidal ideation, attempts, and death by suicide, several gaps in the literature need to be addressed to advance research and improve clinical practice. This Call-to-Action highlights three major gaps in our understanding of the intersection of psychosis and suicide as determined by expert consensus. The three gaps include research methods, suicide risk screening and assessment tools used with persons with psychosis, and psychosocial interventions and therapies. Specific action steps to address these gaps are outlined to inform research and practice, and thus, improve care and prognoses among persons with psychosis at risk for suicide.

## 1 Introduction

Persons with schizophrenia spectrum disorders have an increased risk of suicide, up to 20 times that of the general population (1–3). While the risk of suicide is particularly elevated in the first episode and early stages of psychosis, it remains a major concern throughout the course of illness (4), with the lifetime rate of death by suicide estimated to range between 4 to 13% (5–7). According to a recent meta-analysis, individuals in the general population who self-report psychotic experiences are twice as likely to present with subsequent suicide ideation than the general population, three times as likely to attempt suicide, and four times as likely to die by suicide (8). Relatedly, approximatively 30-50% of individuals with schizophrenia spectrum disorders or affective disorders with psychotic features will have suicide ideation in their lifetime (9–11) and 20-50% will attempt suicide (7, 12, 13) highlighting the impact of suicide risk among those with psychosis.

While there has been substantial progress in research and clinical care for individuals experiencing psychosis with suicide risk, gaps remain. Notably, there are three major gaps identified determined by expert consensus (14) that deserve attention: 1) lack of research design standards, 2) unknown reliability and validity of suicide risk screening and assessment tools in a psychosis population, and 3) insufficient evidence of efficacious suicide-focused psychosocial interventions and therapies for those experiencing psychosis. We outlined several action steps that we believe will help researchers and providers fill these gaps.

## 2 Implement research design standards that consider the heterogeneity of both psychosis and suicide thoughts and behaviors to support data aggregation

Both suicide risk and psychosis encompass a wide range of definitions and broad continuum of severity (**Table 1**), contributing to unresolved variability in findings across studies. For example, a recent meta-analysis showed that the prevalence of suicide attempts varies according to the specific diagnosis of the schizophrenia spectrum disorder, the setting (outpatient vs. inpatient), and geographical region (23). A better understanding of these consistencies and inconsistencies is an important step in further the translation of this research into effective clinical intervention. Furthermore, in every stage of the research process possible, including research design, delivery, and implementation, researchers should include individuals with lived experience of psychosis and suicide risk (24–26). This may also include the acknowledgement that many researchers have lived experience themselves. The inclusion of people with lived experience will improve the quality and relevance of research to those who are most impacted: those seeking services. Finally, it is important to acknowledge the challenges of recruiting and retain participants with psychosis in research and how these challenges impact data ascertainment, e.g., (27). We therefore present recommendations for implementing research design standards on suicide risk in psychosis, while considering their heterogeneity and the aforementioned factors.

**Table 1:** Term Definitions.

Suicide	A death caused by a self-directed injurious behavior with some intent to die from that behavior (15)
Passive suicide thoughts	Referring to a desire to be dead (16)
Active suicide ideation	Referring to having a thought of wanting to kill oneself with or without a plan (15)
Suicide attempt	Defined as non-fatal, self-directed, and potentially injurious behavior with a degree of intent to die from that behavior, which may or may not involve injury (15)
Psychosis symptoms	Positive symptoms, i.e., hallucinations, delusions, and disorganized behavior, and negative symptoms, e.g., affective flattening, alogia, avolition, anhedonia, and asociality (17)
Schizophrenia spectrum or other psychotic disorder diagnosis	Diagnoses of schizophrenia spectrum or other psychotic disorders, e.g., diagnoses of schizophrenia, schizoaffective disorder, schizophreniform disorder, brief psychotic disorder, delusional disorder, schizotypal personality disorder (17)
Affective psychosis	Psychosis symptoms occur as part of a mood disorder with psychotic features, i.e., bipolar I disorder with psychotic features or major depressive episode with psychotic features (17)
Clinical-high risk for psychosis, also called ultra-high-risk, at-risk, prodromal phase	Persons at clinical high-risk for psychosis are deemed at risk of transitioning to overt psychosis, and typically present with attenuated symptoms of psychosis, brief and limited intermittent episodes of psychosis, disturbing experiences in different domains, e.g., perceptions, thought processing, attention, or a decline in their general functioning with a history of psychosis in the family (18–21)
First-episode psychosis	The onset of the first episode of psychosis is usually marked by the presentation of frank symptoms of psychosis and the fulfillment of diagnostic criteria for a schizophrenia spectrum disorder or an affective disorder with psychotic features (22)

### 2.1 Recruit diverse samples across the psychosis spectrum

#### 2.1.1 Include individuals experiencing varying levels of psychosis severity and diagnoses

Researchers should aim to recruit samples of people along a spectrum of psychosis symptom severity. This would include those with self-reported psychotic experiences in the general population who meet ultra or clinical high-risk criteria or who have experienced a first episode of psychosis, to those who have had a chronic schizophrenia spectrum disorder or disorder with psychotic features for many years. The needs and characteristics of young adults with a first episode of psychosis are different from those of people who have been living with the illness for several years, including in terms of stage of development and expectations of treatment (28, 29). It is therefore possible that suicide risk factors, as well as effective suicide prevention treatments, differ across people at different stages of illness.

#### 2.1.2 Recruit participants from a variety of community and clinical settings

The clinical profile of patients may vary depending on the community or clinical setting (e.g., inpatient units, outpatient services, specialized services for psychosis, such as early intervention services), and certain protective factors may also influence suicidal behaviors (e.g., safety measures during hospitalization (23). Studies should recruit participants from a variety of settings to ensure that samples include varying levels of suicide risk and psychosis symptom severity.

#### 2.1.3 Recruit and include diverse samples from differing racial and ethnic backgrounds, developing nations, and LGBTQ+ groups

This is especially important given racial and ethnic disparities in psychosis risk across the continuum (30–32) and that risk factors may vary across racial and ethnic groups (33–35). For example, in first generation immigrants and Hispanic/Latinx persons with a schizophrenia spectrum disorder, maintaining cultural traditions from their country of origin, religious beliefs, and practices, and having a positive ethnic identity may be protective against suicide ideation (36, 37). This could translate into specific targets for assessment and suicide prevention interventions in these groups. Ethnicity and race should be carefully assessed (e.g., based on self-identification, multiple data sources, migratory status (35)). Increased culturally sensitive and inclusive research will contribute to the development of culturally sensitive suicide prevention interventions.

Knowledge and evidence on prevalence, risk factors, theoretical models (38, 39) and suicide prevention interventions (40, 41) come predominantly from high-income countries. However, there have been reports of different rates and risk factors for suicide thoughts and behaviors in persons with schizophrenia spectrum disorders between low- and middle-income countries and high-income countries (42–45). A cross-national comparative study revealed that self-reported psychotic experiences were less distressing for adults living in low- and middle-income countries than for those in high-income countries (46). However, it is possible that the cross-cultural validity of current measures of psychotic experiences is limited, that psychotic experiences of equal severity are less clinically relevant in some low- and middle-income countries due to certain social and cultural protective factors (e.g., collectivist communities), or that the association between psychotic experiences and distress, and possibly suicide thoughts and behaviors, differs between countries due to cultural differences (47). Measures of psychotic experiences need to be validated across countries to minimize intra-category variability and refined to focus more on distress to better identify individuals with greater needs among different cultures, notably in terms of suicide screening and prevention (47).

LGBTQ+ groups have a higher risk of suicidal ideation and behavior, owing in part to the unique challenges they may face and minority stressors (48–50), and may be a greater risk for experiencing psychosis (51, 52). It is crucial to better understand the unknown role of LGBTQ+ identities on suicide risk in psychosis as well as providing a broader understanding of the intersection of LGBTQ+ identities and psychosis. Including affirming questions include asking one’s gender identity as well as sex (e.g., 53, 54), can be a step forward to address these questions in future research.

### 2.2 Conduct longitudinal observational studies

Many studies of psychosis and suicide in the literature are cross-sectional (55–57), limiting the conclusions that can be drawn to impact the assessment of suicide risk, suicide prevention interventions, and understanding of mechanisms underlying the association between psychosis and suicide risk (55). While many studies have reported that psychosis is a risk factor for suicide (8, 10), recent evidence suggests a bidirectional relationship between psychosis-like experiences and suicide thoughts and behaviors (58, 59). Further longitudinal studies designed specifically to address these questions of clinical relevance are needed to inform suicide prevention practices. Study designs that combine short-term high intensity measurement and long-term panel type designs (i.e., measurement burst) may be useful to uncover the dynamics of psychosis-like experiences on suicide thoughts and behaviors.

### 2.3 Conduct largescale, multisite research that supports data sharing

Although the risk of suicide is high in persons with psychosis, the lifetime prevalence of all schizophrenia spectrum disorders and affective disorders with psychotic features is estimated at 3.06% (60), and up to half will experience suicide ideation or attempts in their lifetime (7, 9–13). Thus, to conduct studies with sufficient statistical power, largescale, multisite research studies are needed, with large sample sizes to provide a more robust evidence base. Pooling resources from different sites together may be one approach. Fortunately, large data sets already exist across a range of general and clinical populations and countries; more effort can be dedicated to merging these data sets for cross-site analyses. Future research should establish consistent measurement harmonization, which is needed for these analyses. Initiatives, such as the National Institutes of Health (NIH) Data Management and Sharing policy (61), the NIH Data Archive (62), and the National Institute of Mental Health MAP-PRO (Meaningful Assessment Protocol- Patient-reported outcomes) platform (63, 64), are also needed to improve our understanding of relatively rare phenomenon, such as suicide in people with psychosis.

## 3 Determine the reliability and validity of suicide risk screening and assessment tools in psychosis populations

Suicide screeners are used to identify those at risk for suicide (e.g., recent suicide thoughts or behaviors) while assessment tools develop a more comprehensive understanding of that suicide risk (e.g., severity and frequency of suicide thoughts, risk and protective factors). Both are crucial for early identification and prognosis of care. Moreover, using valid and reliable tools are necessary to develop an accurate understanding of suicide risk (65, 66) and an understanding of the unique phenomenology of suicide risk in those experiencing psychosis.

### 3.1 Test the use of established suicide risk screening and assessment tools among those with psychosis

Overall, it is important to be screening and assessing for suicide risk in all populations and studies in the general population have consistently demonstrated that asking about suicide does not increase the risk of suicide (67, 68). In fact, there may be a benefit to asking about suicide (69). Given the increased suicide risk in a psychosis population it may be that much more crucial.

It is possible that existing current suicide risk screening tools are appropriate for individuals with psychosis, but this has yet to be established. Establishing the validity, reliability and utility of existing validated screeners or measures of suicide risk (e.g., the Ask Suicide-Screening Questions (ASQ) Toolkit (70–72)), the Beck Scale for Suicide Ideation (BSS (73, 74)), the Columbia – Suicide Severity Rating Scale (C-SSRS (75, 76)); in people with psychosis will help foster the harmonization and use of gold-standard measures of suicide thoughts and behaviors in this population. Notably, one study found that both the BSS and the C-SSRS were able to collect suicide attempt history for patients with schizophrenia spectrum disorders (77). Measures of suicide thoughts and behaviors, as well as psychosis experiences, should be harmonized across studies, building off broader efforts to standardize assessments to allow direct comparison across research studies. Additionally, future cross-sectional and longitudinal studies should carefully consider the timing of assessments. Many studies assess risk factors long before the suicide outcome occurs or use lifetime measures, which while useful may miss nuance captured by measures that incorporate questions about current suicide ideation (56). Future studies should examine lifetime and current suicide risk-related symptoms to better capture chronic risk factors (e.g., demographics) versus acute risk factors.

### 3.2 Consider the unique aspects of psychosis that may influence the assessment of suicide risk

There are several unique aspects of psychosis and schizophrenia spectrum disorders that may influence the assessment of suicide risk. For some people with psychosis symptoms, psychiatric treatment or involuntary hospitalization can be unpleasant and potentially traumatic (78, 79). Therefore, it is possible that people with psychosis may hesitate to disclose suicide ideation to a provider for fear of further hospitalization. Second, there is some evidence that people with psychosis have impairments in metacognition and awareness of their thoughts (80, 81), which may impact their ability to report on suicide risk. This may be particularly relevant for details of such thoughts (e.g., recency, content). Third, people with psychosis experience both public and internalized stigma related to their mental health diagnoses or symptoms (82–84), which may increase risk of suicide (85, 86). It is possible that the stigma that people with psychosis experience compounds with stigma about suicide risk, further impacting their willingness to disclose suicidal thoughts.

Psychosis impacts the phenomenology of suicide. There is some evidence for greater medically lethal means used in psychosis populations (e.g., firearms (87)) and a longer duration of untreated psychosis is associated with an increased risk of suicide behavior (88). For some individuals with a schizophrenia spectrum disorder, suicide behaviors represent a direct response to command auditory hallucinations (e.g., 89). However, in one study, the rate of suicide attempts did not differ between those who experienced command hallucinations and those who did not, suggesting that other factors are important in identifying individuals at risk of suicide behavior among those with schizophrenia spectrum disorders (89).

### 3.3 Differentiate and assess specific symptoms of psychosis (both positive and negative) in relation to suicide risk, including whether symptoms of psychosis alter the phenomenology of this suicide risk

Given inconsistent findings regarding the relationship between psychosis experiences and suicide thoughts and behaviors, a fine-grained analysis of specific psychosis symptoms (and their severity) with subtypes of suicide thoughts and behaviors (i.e., ideation, plans, attempts, death) could help better understand whether, for example, the association between psychosis and suicide thoughts and behaviors is due to the presence of psychosis symptoms, to specific symptoms (e.g., auditory versus visual hallucinations (90), to the frequency or intensity of these (90), to the associated distress (91), or is mediated by another variable (55). Relatedly, considerations of unique risk factors and protective factors and sources of strengths for this population should be determined and routinely assessed. For example, negative symptoms may be related to reduced suicidal behavior (10, 92) and defeatist attitudes may further be protective from suicide (93).

### 3.4 Understand mechanisms and unique aspects of living with psychosis related to suicide risk

To fully assess for suicide risk in psychosis, understanding specific suicide risk factors and the phenomenology of suicide in this population, and whether these constructs differ from the general population, is crucial. Literature to date suggest the presence of various demographic characteristics (e.g., age, gender, history of suicide attempt), psychiatric symptom experiences (e.g., depression, hopelessness, psychosis symptoms), clinical insight, and substance use disorders contribute to suicide risk in the psychosis population (1, 8, 56, 57, 94–100). There is variability across studies in terms of whether risk factors (1) statistically explain the association between psychotic symptoms and suicide behavior or (2) whether psychotic symptoms themselves are an independent risk factor for suicide risk, adjusting for these factors (55, 101).

## 4 Determine effective psychosocial interventions and treatments that directly target suicide risk among persons with psychosis

Psychosocial interventions and treatments to address suicide outcomes among psychosis populations are limited. (Note. We do not address pharmacologic treatments in this review but acknowledge that many gaps exist in that treatment domain as well.) A recent systematic review and meta-analysis examined 11 studies of psychosocial interventions among participants with psychosis with measurement of suicide outcomes and found a significant treatment effect that pooled across suicide ideation, attempt, and death (7). Interventions of the 11 studies included various approaches (i.e., supportive treatment, cognitive-behavioral, cognitive, case management) compared to treatment as usual or a waitlist, with cognitive-behavioral therapy (CBT) being the most prevalent approach (7). While the meta-analytic study by Bornheimer and colleagues (2020) had potential methodological limitations associated with selection bias due to the requirement of suicide-related outcomes being measured in the studies examined, the study provides preliminary support for psychosocial interventions in psychosis that captured suicide ideation, attempt, and death. Importantly, it also highlights the lack of empirical studies investigating their effect and the lack of consensus on best practices due to the wide variety of intervention characteristics in the included studies (7). Further gaps remain as there is little understanding of interventions directly targeting suicide thoughts and behaviors in psychosis. Moreover, and relatedly, the potential moderators of treatment uptake and retention are unknown, which may include more severe psychosis (e.g., 27).

### 4.1 Test the use of suicide prevention interventions across the psychosis spectrum

Aside from studies who focus on suicide and psychosis within their research, many trials investigating suicide-related outcomes often exclude psychosis populations, and vice-versa (e.g., 102–107). For example, a recent systematic review of psychotherapy trials with suicide-related primary outcomes found that 75% excluded individuals with psychosis (102). Given this exclusion rate, this review highlights that there is a dearth of information on if suicide prevention interventions are effective for those with psychosis. In the review, few studies provided a rationale for excluding people with psychosis (102). Therefore, existing suicide-focused interventions (e.g., Brief Cognitive-Behavioral Therapy for suicide prevention (BCBT (108, 109)), Cognitive Therapy for Suicide Prevention (CT-SP (110)), Collaborative Assessment and Management of Suicidality (CAMS (111)), crisis lifelines (112), lethal means counseling ((113), safety planning type interventions (114)) should be evaluated in psychosis, and if needed, modifications and adaptations should be made to make these interventions more acceptable or effective. Furthermore, trials should provide a rationale for why psychosis is excluded so that interventions could be developed to reduce barriers (e.g., support for decisional capacity to consent to research).

### 4.2 Test novel suicide prevention intervention adaptations across the psychosis spectrum

It is possible that existing suicide prevention interventions do not require modification for use with people with psychosis, but in the absence of specialized research, licensed practitioners may consider how suicide prevention interventions may need to be adapted due to unique facets of working with this population. For instance, people who have a schizophrenia spectrum disorder may experience cognitive impairments (115–119), so compensatory strategies may help with intervention retention and skill use. Additionally, positive psychosis symptoms such as hallucinations may be indicators of greater risk for suicide thoughts or behaviors (10, 120, 121), and their assessment should be incorporated into interventions if applicable. Furthermore, people with psychosis may have limited social supports (122–125). Lacking social connection may be associated with increased suicidal desire (e.g., thwarted belongingness and perceived burdensomeness (126)) and a higher risk for future suicide attempt or death if social contacts are not listed on their suicide safety plan (127). People with psychosis may be less likely to use crisis lines (128) and interventions exist to practice and gain exposure to crisis line calls and safety planning may need to be augmented (e.g., 129). See examples of interventions that have explicitly been adapted for psychosis in **Table 2**.

**Table 2:** Examples of suicide-focused interventions that have explicitly been adapted for psychosis.

Intervention Name	Short Description	Research Stage
Acceptance and Commitment Therapy (ACT): Creating a Life Worth Living	A 60 minute, 8-week group therapy utilizing concepts of ACT and safety planning.	A pilot feasibility and acceptability study was completed with promising outcomes with Veterans in a VHA psychosocial rehabilitation program (130).
Cognitive-Behavioral Suicide Prevention for psychosis (CBSPp)	CBSPp uses cognitive and behavioral techniques to identify and modify information processing biases, appraisals, and schemas related to suicide and psychosis.	A pilot randomized controlled trial found CBSPp to be feasible with positive outcomes (131). Adaptations for community mental health are ongoing (132) with promising significant open pilot trial findings (133).
SafeTy And Recovery Treatment (mSTART)	A cognitive behavioral therapy intervention with a mobile device to support in-between session learning to reduce suicide risk among people with schizophrenia and bipolar disorder.	A pilot trial of mSTART found promising feasibility, acceptability, and significant improvement in suicide ideation severity (27, 134)
Suicide Prevention by Peers Offering Recovery Tactics (SUPPORT) Program	A recovery-oriented suicide prevention intervention delivered by Veteran Peer Specialist to a Veteran with serious mental illness at risk for suicide in four, 50-minute individual appointments. Includes standalone suicide prevention training and a training specific to delivering the intervention for Certified Peer Specialists.	An open pilot trial to determine feasibility, acceptability, and fidelity is underway at a VHA medical center (135).
Youth-Nominated Support Team (YST) Intervention	A psychoeducation, support-based intervention for adolescents to nominate an adult who in turn provides support to the adolescent at risk for suicide, typically for three months following hospitalization.	The program is being adapted for clinical-high risk for psychosis with suicidal ideation (136).

VHA, Veterans Health Administration.

## 5 Conclusion

Suicide is a critical public health issue among individuals with psychosis. Although progress has been made in research and clinical care for individuals at risk for suicide and experiencing psychosis, important gaps remain. This paper presented three major areas for attention and action, including: 1) implement research design standards that consider the heterogeneity of both psychosis and suicide thoughts and behaviors, 2) determine the impact and utility of suicide risk screening and assessment tools in psychosis populations, and 3) determine effective psychosocial interventions and therapies that directly target suicide risk among persons with psychosis. In addition to the above recommendations, there are two additional points we would like to highlight. First, funding agencies should seek to increase funding opportunities at the intersection of suicide and psychosis to support critically needed research in these areas to impact practice and policy. Second, trainings should be developed, tested, and implement to prepare providers with competency and effectiveness in treating suicide risk and psychosis. Overall, the action steps provided in the areas above aim to provide a framework for addressing these critical gaps in research.

## Data availability statement

The original contributions presented in the study are included in the article/supplementary material. Further inquiries can be directed to the corresponding author.

## Author contributions

SC: Conceptualization, Funding acquisition, Investigation, Methodology, Supervision, Writing – original draft, Writing – review & editing. RS: Conceptualization, Writing – original draft, Writing – review & editing. LB: Writing – review & editing, Writing – original draft, Conceptualization. EP: Conceptualization, Writing – original draft, Writing – review & editing. HW: Writing – original draft, Writing – review & editing. BE: Writing – review & editing, Writing – original draft. JD: Writing – original draft, Writing – review & editing. CD: Writing – original draft, Writing – review & editing.
